# Cure for Leprosy

**Published:** 1915-06

**Authors:** 


**Cure for Leprosy.** According to the Texas Med. Jour., May 1915, Dr. Alph H. Boehmer, Surgeon General of Siam, claims that the disease is curable and, as is well established, that the actual danger of contagion is slight.

